# First report of three complete mitochondrial genomes of the long-finned charr *Salvethymus svetovidovi* Chereshnev et Skopetz, 1990 (Salmoniformes: Salmonidae) with phylogenetic consideration

**DOI:** 10.1080/23802359.2019.1638325

**Published:** 2019-07-13

**Authors:** Alla G. Oleinik, Lubov A. Skurikhina, Andrey D. Kukhlevsky, Alexander A. Semenchenko

**Affiliations:** a A. V. Zhirmunsky Institute of Marine Biology, National Scientific Center of Marine Biology, Far Eastern Branch, Russian Academy of Sciences, Vladivostok, Russia;; b Far Eastern Federal University, Vladivostok, Russia

**Keywords:** Charr genus *Salvelinus*, salmonids, Svetovidov’s long-finned charr, *Salvethymus svetovidovi*, mtDNA, mitogenome, phylogeny

## Abstract

The complete mitochondrial genome was sequenced in three individuals of long-finned charr *Salvethymus svetovidovi* from Lake El’gygytgyn (Chukotka Peninsula, Russia). The genome sequences are 16,655 bp in size and the gene arrangement, composition, and size are similar to the charr genomes published previously. The difference between the three genomes studied is low, 0.07%. Our results support the phylogenetic closeness of *Sl. svetovidovi* with representatives of the genus *Salvelinus* and their origin from a common ancestor. A placement of *Sl. svetovidovi* in the phylogenetic tree is strictly defined and this taxon should be included in the genus *Salvelinus.*

The long-finned charr *Salvethymus svetovidovi* Chereshnev et Skopetz, 1990 is a unique endemic narrow-range species represented by a single population in ancestral Lake El’gygytgyn (Chereshnev et al. 2002). The peculiarity of *Sl. svetovidovi*is is associated with the specific features of the lake, located to the north of the Chukotka Peninsula, Russia (67°30′ N/172°05' W) at an altitude of 490 m above sea level. Lake origin (about 3.6 Mya) is attributed to a meteorite impact or to a gas explosion of endogenous nature. Originally, this taxon was separated into a new monotypic distinct genus based on unique morphological and ecological characters (Chereshnev and Skopets 1990). It was suggested that *Sl. svetovidovi* is one of the most ancient salmonid taxa phylogenetically close to the common ancestor of charr of the genus *Salvelinus* (Chereshnev and Skopets 1990). Karyological analysis generally confirmed this suggestion. *Sl. svetovidovi* has a unique karyotype, which evolved owing to Robertsonian translocations independently from the species of the genus *Salvelinus*, though preserving some characters in common with charr (Frolov 2000). The hypothesis subsequently caused a broad discussion. More recently, it was shown that the long-finned charr *Sl. svetovidovi* possesses a mosaic combination of morphological and karyological characters typical of both phylogenetically advanced and archaic salmonid taxa, suggesting deep specialization (Stearley and Smith 1993; Chereshnev et al. 2002). In addition, alternative hypotheses were advanced that require revision of the current views on the ancient origin and taxonomic status of *Sl. svetovidovi* (Alekseyev 2000; Osinov et al. 2015). However, little genetic information of *Sl. svetovidovi* is available. Currently, genetic evidence is insufficient to correctly analyze the origin and relationships of *Sl. svetovidovi*. Most of the previous studies of this species are restricted to an analysis of only parts of single mitochondrial genes (mostly control region) (Brunner et al. 2001) or a few mitochondrial and nuclear genes (Crete-Lafreniere et al. 2012; Osinov et al. 2015). Therefore, based on the previous studies, the phylogenetic position of the genus *Salvethymus* was not well resolved.

To overcome these limitations, we have sequenced three complete mitochondrial genomes of *Sl. svetovidovi* for the first time. The fish specimens are stored in the collection of the Genetics Laboratory, National Scientific Center of Marine Biology FEB RAS, Vladivostok, Russia (www.imb.dvo.ru) under accession SVET17.001, SVET17.002, and SVET17.004. Totally, 5 pairs of primers were used (sequences are available upon request), which were designed based on public mitochondrial genome sequences available in GenBank for salmonid fishes. The sequenced fragments were *de novo* assembled into the complete mitochondrial genome and annotated by comparing with published genome sequences of other charr using Geneious R11 (http://www.geneious.com/). To confirm the phylogenetic position of *Sl. svetovidovi*, 17 mitochondrial genomes of representatives of genus *Salvelinus* together with *Parahucho* and *Salmo* as outgroups were aligned with the MAFFT algorithm in Geneious, and maximum likelihood (ML) analysis based on the Tamura-Nei (TrN93) plus gamma model of nucleotide substitution was conducted. The ML tree was constructed using the MEGA X software (Kumar et al. 2018) and validated by the bootstrap analysis. Finally, a physical map of *Sl. svetovidovi* mitochondrial genomes was generated and uploaded to GenBank with accession numbers MK695627, MK695628, and MK695629.

The complete mitochondrial genome of native *Sl. svetovidovi* was 16,655 bp in length. The genomic organization was identical to those of typical salmon mitochondrial genomes, including 2 rRNA genes, 13 protein-coding genes, 22 tRNA genes, a light-strand replication origin (OL), and a control region (CR). Like the charr mitochondrial genomes (Balakirev et al. 2016), the overall base composition was 28.0% of A, 26.4% of T, 28.7% of C, and 16.9% of G with a slight A + T bias (54.5%). We detected 18 single-nucleotide and no any length differences between the sequences MK695627, MK695628, and MK695629**;** only 13 substitutions were found in overall protein coding sequences and five were detected in control region. The proportion of variable sites was highest for the NADH dehydrogenase subunit genes (55.6%). Total sequence divergence (*D_xy_*) was 0.0007 ± 0.0001.

The comparison of mitochondrial genomes now obtained with other complete mitochondrial genomes of related groups available in GenBank including genera *Salvelinus* (GenBank accession numbers AF154851, KF974451, KJ746618, KJ746619, KT266870, KT266871, KU674351, KU674352, MK695630, MK695631, NC000860, NC036392, and NC037502), *Salmo* (AF133701, and AM910409), and *Parahucho* (KJ816315, and KJ816316) revealed a close affinity of *Sl. svetovidovi* to the *Salvelinus* species (Figure 1). In the case of the genera *Salvelinus*, *Parahucho, and Salmo*, the phylogenetic relationships among species were similar to those inferred in Balakirev et al. (2016). Our data unambiguously point to the independent taxonomic status of *Sl. svetovidovi* but within the genus *Salvelinus*. The difference (*D_xy_*) between them was 0.0234 ± 0.0012, which is in close agreement with the values of interspecific divergence previously reported for the charr (e.g. Oleinik et al. 2015 and references therein). The lowest sequence divergence (*D_xy_* = 0.0166 ± 0.0009) was detected between our specimens of *Sl. svetovidovi* and the complete mitochondrial genome of *S. taranetzi* (MK695630, and MK695631). The difference (*D_xy_*) between *Salvethymus* and *Parahucho, Salvethymus,* and *Salmo* was 0.0938 ± 0.0022 and 0.1047 ± 0.0024. Moreover, *Salvethymus* and *Salvelinus* showed very similar levels of divergence with those of *Salmo* and *Parahucho* representatives (0.0988 ± 0.0022 on average).

**Figure 1.**
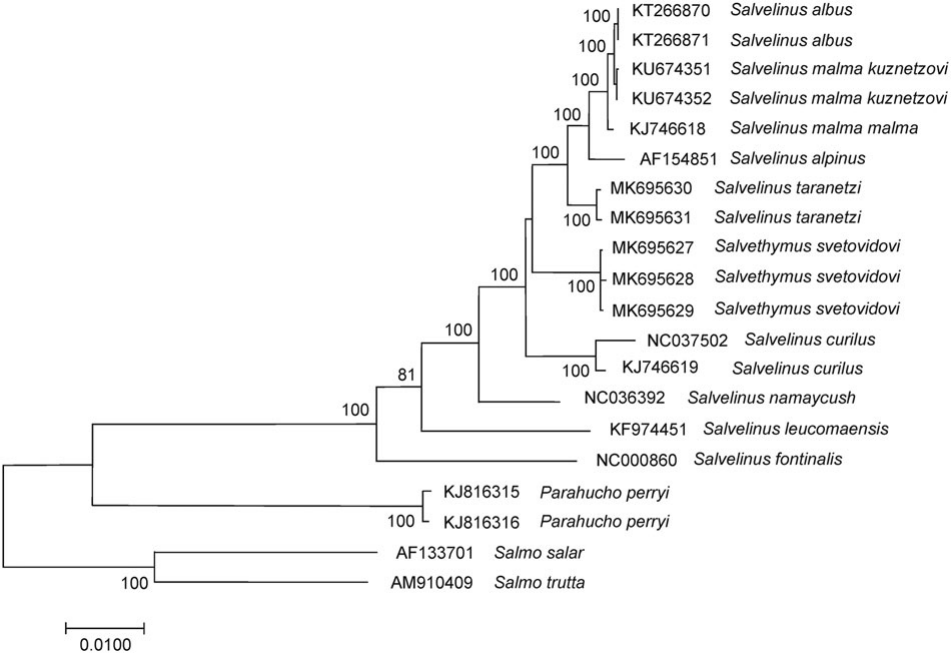
Maximum likelihood tree constructed based on the comparison of complete mitochondrial genome sequences of *Salvethymus svetovidovi* specimens and other GenBank representatives of the family Salmonidae. The tree is based on the TrN93 plus gamma model of nucleotide substitution. Genbank accession numbers for all sequences are listed in the figure. Numbers at the nodes indicate bootstrap probabilities from 1000 replications (values below 80% are omitted).

Charr of the genus *Salvelinus,* including *Salvethymus,* represent a monophyletic group of salmonid fishes. Based on the variability of complete mitochondrial genomes, *Sl. svetovidovi* was positioned on the phylograms as a sister group to cluster (*S. taranetzi* ((*S. malma malma*, *S. malma kuznetzovi, S. albus*) *S. alpinus*)). Our results refute the phylogenetic hypotheses which suggest the early divergence of *Sl. svetovidovi* from the charr common ancestor and support previous findings that genus designation is not required for *Salvethymus*.

Analysis of the complete mitochondrial genomes from diverse species of charr would give insight into molecular phylogenetic relationships and help improve the understanding of historical and taxonomic relationships from previous morphological and ecological studies (Chereshnev et al. 2002; Alekseyev 2000). *Sl. svetovidovi* is the most striking example of adaptation during isolation. This taxon has acquired unique morphological features as a result of specialization. The observed noncorrespondence of morphological (Chereshnev et al. 2002) and genetic differentiation (Figure 1) can be explained by uneven evolutionary dynamics of qualitatively different features among exceptional ecological plasticity of charr.
